# Parental Satisfaction With Pediatric Dental Care Provided by Undergraduate Students at King Saud bin Abdulaziz University for Health Sciences

**DOI:** 10.7759/cureus.88899

**Published:** 2025-07-28

**Authors:** Asma A Alshahrani, Haifa A Alamro, Salman M Alhilali, Meshal A Alwadi, Nada A Alshahrani, Ali K Asiri

**Affiliations:** 1 Preventive Dental Science Department, College of Dentistry, King Saud Bin Abdulaziz University for Health Sciences, Riyadh, SAU; 2 Research Department, King Abdullah International Medical Research Center, Riyadh, SAU; 3 Prosthodontics Department, Ministry of National Guard-Health Affairs, Riyadh, SAU; 4 College of Dentistry, King Saud Bin Abdulaziz University for Health Sciences, Riyadh, SAU; 5 Epidemiology and Public Health Research Department, University College London, Riyadh, SAU; 6 Pediatric Dentistry and Orthodontics Department, King Saud University, College of Dentistry, Riyadh, SAU

**Keywords:** dental education, parental satisfaction, pediatric dentistry, saudi arabia, undergraduate dental students

## Abstract

Background: Pediatric dental care emphasizes the holistic well-being of children, integrating physical, psychological, and social aspects of their health. Parental satisfaction serves as a valuable indicator of healthcare quality, influencing children’s adherence to dental visits and long-term oral health outcomes.

Objectives: This cross-sectional study aimed to assess parental satisfaction with pediatric dental care provided by undergraduate dental students at the College of Dentistry (COD), King Saud bin Abdulaziz University for Health Sciences (KSAU-HS), Riyadh, Saudi Arabia.

Methods: A validated, interviewer-administered questionnaire was used to collect data from 128 parents of children aged 12 years or younger who received dental treatment between January and May 2023. The questionnaire addressed demographic information, appointment details, and satisfaction using a five-point Likert scale.

Results: A total of 128 parents or primary caregivers participated in the study. Most were mothers (62.5%) and aged between 35 and 44 years (57.8%). The majority (99.2%) reported that they were either satisfied with the pediatric dental care their child received. Overall satisfaction was significantly associated with waiting time (p = 0.002) and reason for visit (p = 0.042). No significant associations were found with parental age, education level, or student year.

Conclusion: Ongoing assessment of parental satisfaction can inform improvements in clinical education and patient care within undergraduate dental programs.

## Introduction

Pediatric dental care embraces the concept of a child's physical, psychological, and social well-being [1]. Access to oral healthcare for children is a critical consideration that involves both the availability and utilization of care [2]. This accessibility to quality oral healthcare has been intricately based on sociological, economic, and geographical variables [3]. Acknowledging parental satisfaction underscores its crucial role as a key metric for assessing the oral quality of care provided [1,2]. Additionally, understanding parental perspectives enhances the approach, benefiting both young patients and families, and creating an enriching environment for pediatric dental care.

Patient satisfaction is determined by a combination of patient expectations and perception of the service provided. Surveys for measuring patient satisfaction are widely used in medical care evaluation worldwide [4]. They can demonstrate the outcomes of provided healthcare based on patients’ feedback. The content of these measures evaluates the main factors that influence patient satisfaction, which can aid in addressing the strengths and weaknesses of dental services and improving treatment quality. It has been previously observed that satisfied patients are more inclined to build a positive relationship with the healthcare system, resulting in enhanced compliance, continuity of care, and improved health outcomes [5].

Parental satisfaction in pediatric dental care may lead to improved long-term oral health outcomes. The effect of this satisfaction would encourage parents to bring their children to regular check-ups, receive necessary treatments, and adopt appropriate oral hygiene practices. This approach significantly decreases the future risk of serious dental problems and fosters a positive attitude toward dental care. Moreover, ensuring an oral healthcare system that meets parental expectations will enhance the healthcare environment for the child.

Several studies have been carried out to assess patient satisfaction in centers that provide oral healthcare, particularly in terms of clinical quality, facility conditions, and accessibility [6-8]. However, there has been limited research on parental satisfaction with dental care provided to their children by dental students. Therefore, the aim of this study is to assess parental satisfaction with pediatric dental care provided by undergraduate dental students at the College of Dentistry (COD), King Saud bin Abdulaziz University for Health Sciences (KSAU-HS), Riyadh.

In addition, the study’s objectives are to examine associations between parental satisfaction and variables such as waiting time, student level, and reason for visit; report levels of parental satisfaction across different aspects of care, such as communication, facility cleanliness, and student professionalism; and provide evidence to guide improvements in both clinical education and patient-centered care in pediatric dentistry training programs.

## Materials and methods

This cross-sectional study was conducted at the COD, KSAU-HS, Riyadh, Saudi Arabia, between January and May 2023. The objective was to assess parental satisfaction with pediatric dental care delivered by undergraduate dental students. Ethical approval was obtained from the Institutional Review Board at King Abdullah International Medical Research Center (IRB/2787/22). Written informed consent was obtained from all participants. Anonymity and confidentiality of the data were assured throughout the study by assigning each participant a unique code number instead of using personal identifiers. No names or identifiable information were recorded on the survey forms, and all data were securely stored in password-protected digital files accessible only to the research team.

Study population and sampling

The study targeted parents or primary caregivers of children aged 12 years or younger, inclusive, who received dental treatment from undergraduate students at the COD, KSAU-HS. Eligible children were classified according to the American Society of Anesthesiologists (ASA) Physical Status Classification System as Class I (healthy) or Class II (mild systemic disease). This classification ensured that all participants were medically stable and appropriate candidates for outpatient pediatric dental care.

Although the inclusion criteria permitted children as young as two years old, the final sample comprised children aged five to 12 years. This distribution reflected the typical patient population treated in undergraduate pediatric clinics, where children under age 5 are infrequently scheduled due to behavioral management considerations and institutional protocols that often refer younger children to postgraduate providers.

The target population was estimated at approximately 200 pediatric dental patients per academic year treated in the undergraduate pediatric clinics. A minimum sample size of 125 participants was calculated using Wayne’s formula for finite population correction [9], assuming a 69% parental satisfaction rate based on prior literature [10] and a 95% confidence level. 

Participants were recruited via convenience sampling from the pediatric dental clinics at the KSAU-HS. After their child’s appointment, eligible caregivers were approached in person by a trained researcher and invited to participate. Informed consent was obtained on-site before administering the survey. Relatives or siblings who accompanied the child but were not the primary caregiver were excluded from the study. Of the 151 eligible caregivers approached, 128 agreed to participate, yielding a response rate of 84.76%.

Survey Instrument

A structured questionnaire consisting of 30 items was used for data collection. The instrument was adapted from a previously published and validated questionnaire developed by Balhaddad et al. [11], with only minor modifications made to suit the local clinical and cultural context. The questionnaire was organized into three main sections:

Demographic section (10 items): Included the child’s and parents’ age and gender, nationality, household income, educational level, and relationship to the child.

Appointment and service details (9 items): Captured information regarding the reason for the dental visit, the type of procedure performed, waiting time, and the presence and involvement of supervising faculty.

Parental satisfaction scale (11 items): Assessed satisfaction using a five-point Likert scale (1 = strongly disagree to 5 = strongly agree), focusing on factors such as ease of appointment scheduling, facility cleanliness, communication, student professionalism, and perceived treatment outcomes. Last item “I am overall satisfied with the visit” was used as the primary outcome measure of satisfaction. 

While several items assessed the broader dental care experience (e.g., cleanliness, waiting area), the satisfaction scale primarily emphasized aspects related to care provided by undergraduate dental students, such as communication, professionalism, and treatment outcomes.

Instrument validation and pilot testing

Although the questionnaire was adapted from a previously validated source [11], content validity was reassessed by five experts in pediatric dentistry and dental public health using Aiken’s V index, a recognized method for evaluating item-level validity [12]. Each item was rated for relevance and clarity, and only items with a score of V ≥ 0.80 were retained. While content validity was addressed, internal consistency reliability (e.g., Cronbach’s alpha) was not recalculated, as only minor modifications were made and the original tool had demonstrated adequate psychometric properties. To ensure usability and clarity, a pilot test was conducted with 10 participants (who were excluded from the main analysis). Based on their feedback, minor wording and formatting adjustments were implemented to improve the instrument’s flow and comprehensibility.

Data collection procedure

Surveys were administered face-to-face by three trained dental interns fluent in both Arabic and English. Training included orientation on research ethics, neutral interviewing, and data recording procedures. Interviews were conducted in the clinic reception and waiting areas. Each session lasted approximately 5-8 minutes. Participation was voluntary and anonymous. Since data collection was conducted in person and each questionnaire was reviewed upon completion, no missing responses were recorded in the dataset.

Statistical analysis

Data were coded and entered into IBM SPSS Statistics for Windows, Version 29 (Released 2021; IBM Corp., Armonk, New York, United States). Demographic characteristics and satisfaction items were analyzed using descriptive statistics (frequency and percentage). For inferential analysis, responses were dichotomized into "Satisfied" (agree or strongly agree) and "Not Satisfied" (neutral, disagree, strongly disagree) for the item “I am overall satisfied with the visit.”

Fisher’s exact test was applied to evaluate associations between parental satisfaction and independent variables such as student level, wait time, and reason for visit, due to violations of normality assumptions. A p-value < 0.05 was considered statistically significant, and all tests were conducted at a 95% confidence interval. 

## Results

The demographic data of the parents are presented in Table 1. A total of 128 parents participated in the study (48 fathers and 80 mothers), with 95% being Saudi nationals. The majority of parents were aged between 35 and 44 years (57.81%). Approximately 86% held a high school degree or higher, and 52.34% were employed. The average monthly household income was 10,000 Saudi Riyals. The children had a mean age of 8.2 years (SD = 1.8). Among the children, 74 were girls and 54 were boys.

**Table 1:** Demographic data of the parents

Variables	Frequency		Percentage
The accompanying parent			
Father	48		37.50%
Mother	80		62.50%
Nationality			
Saudi	122		95.31%
Non-Saudi	6		4.69%
Parent’s age			
18-25	3		2.34%
26-34	34		26.56%
35-44	74		57.81%
45-54	16		12.50%
55-66	1		0.78%
Educational level			
Uneducated	3		2.34%
Primary school	6		4.69%
Intermediate school	9		7.03%
High school	43		33.59%
Bachelor’s degree	61		47.66%
Higher education	6		4.69%
Monthly income			
<5000	25		19.53%
5000-10000	56		43.75%
10000-20000	37		28.91%
>20000	10		7.81%
Employment status			
Unemployed	55		42.97%
Employed	67		52.34%
Retired	6		4.69%
Child’s age			
Five	4		3.10%
Six	22		17.20%
Seven	23		18%
Eight	29		22.70%
Nine	19		14.80%
Ten	16		12.50%
Eleven	11		8.60%
Twelve	4		3.10%
Child’s gender			
Female	74		57.81%
Male	54		42.19%

The distribution of responses related to appointment-related variables is presented in Table 2. Of the 128 cases, 74 operators (58%) were senior students (D4), and 54 (42%) were junior students (D3). Moreover, 71.87% of the participants reported that it took them less than 30 minutes to arrive at the college, while 22.66% took between 30 and 60 minutes. Participants came to the college for the following reasons: 39.84% came to continue planned treatment, 35.94% for tooth decay, 16.41% for full dental check-up, and 7.81% for other reasons. In addition, Table 2 shows that the majority of parents (89.06%) reported that the clinical supervisor was sufficiently present during their child's visit.

**Table 2:** Distribution of answers related to appointment-related variables *Out of 25 pulp therapy cases, only 10 teeth received SSC in the same visit

Variables	Frequency	Percentage
Student’s level		
D3	54	42.19%
D4	74	57.81%
How long did it take to arrive at the college?		
<15 min	41	32.03%
15-30 min	51	39.84%
30-60 min	29	22.66%
1-2 hours	6	4.69%
>2 hours	1	0.78%
Reason for the visit		
Full dental check-up	21	16.41%
Tooth decay	46	35.94%
Swelling	1	0.78%
Pain	5	3.91%
Dental trauma	1	0.78%
Teeth alignment concerns	3	2.34%
Continue planned treatment	51	39.84%
Treatment provided		
Treatment plan	81	63.28%
Prophylaxis and fluoride	26	20.31%
Tooth filling	51	39.84%
Pulp therapy	25*	19.53%
Stainless steel crown	28	21.88%
Tooth extraction	34	26.56%
Waiting time		
Seen on time or earlier	79	61.72%
15 min	23	17.97%
15-30 min	10	7.81%
30-60 min	10	7.81%
>60 min	2	1.56%
Cannot remember	4	3.13%
Student’s supervisor		
Sufficiently present	114	89.06%
Seen once	11	8.59%
Never seen	3	2.34%
Time preference		
Morning (9 am-11:30 am)	19	14.84%
Afternoon (1 pm-3:30 pm)	102	79.69%
No preference	7	5.47%
How did you hear about COD?		
Family and friends	114	89.06%
Advertisement	4	3.13%
Another health center	3	2.34%
Social media	3	2.34%
Others	4	3.13%
Why did you choose the dental clinics in the college?		
Good quality treatment	73	57.03%
Easy access for the emergency service	9	7.03%
Free service	22	17.19%
Short inter-appointment time	20	15.63%
Others	4	3.13%
How likely are you to recommend our service?		
Extremely likely	108	84.38%
Likely	14	10.94%
Neutral	3	2.34%
Unlikely	2	1.56%
Extremely unlikely	1	0.78%

**Figure 1:**
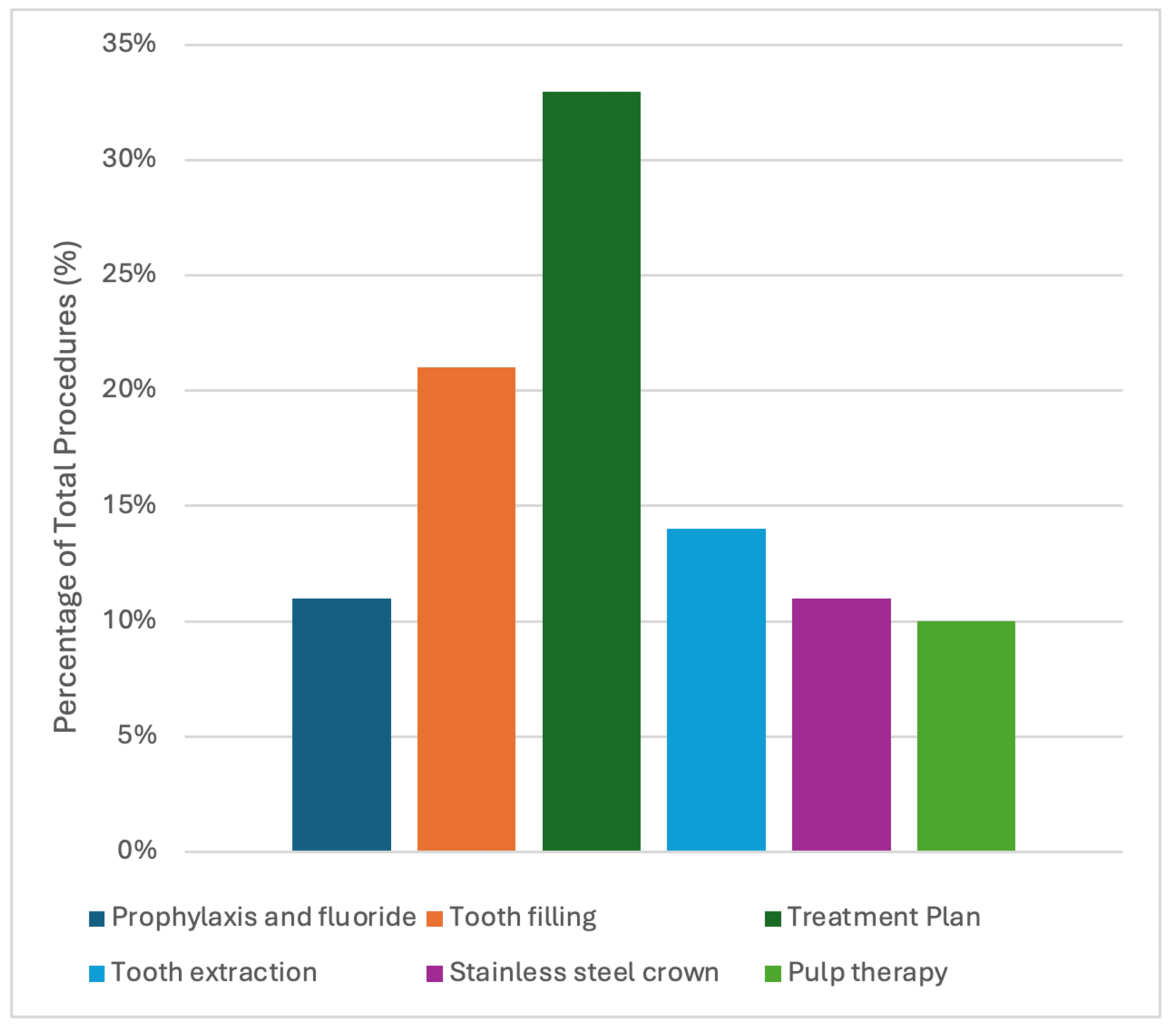
Frequency distribution of performed pediatric dental procedures out of 245 provided procedures

Procedures performed during the visit included treatment planning, prophylaxis and fluoride application, tooth filling, pulp therapy, stainless steel crown placement, and tooth extraction, as presented in Table 2. A total of 245 procedures were completed during the study period. As shown in Figure 1, the most common was treatment planning, with other procedures ranging between 10% and 21%. Furthermore, out of the 25 pulp therapy cases, only 10 received stainless steel crowns in the same visit, as indicated in Table 2.

A high level of parental satisfaction was reported regarding appointment booking, treatment, and facilities at the COD, KSAU-HS. Specifically, 92.9% of parents found the appointments feasible, 96% appreciated the accessibility of the college, and 94% were satisfied with the waiting time (Table 3).

**Table 3:** Distribution of parental responses to individual satisfaction items regarding pediatric dental care (n = 128) Each row presents parental responses to individual satisfaction statements using a five-point Likert scale ranging from “Strongly disagree” to “Strongly agree.” Values are presented as frequency counts with corresponding percentages of the total sample (n = 128)

Statement	Strongly agree	Agree	Neutral	Disagree	Strongly disagree
It was easy to make my child’s first appointment	74 (57.81%)	45 (35.16%)	4 (3.13%)	5 (3.91%)	0
College location was easy to access	81 (63.28%)	42 (32.81%)	3 (2.34%)	1 (0.78%)	1 (0.78%)
The waiting room was clean and neat	84 (66.67%)	37 (29.37%)	4 (3.17%)	1 (0.79%)	0
Enough and clean toilets are available	70 (55.12%)	45 (35.43%)	7 (5.51%)	3 (2.36%)	2 (1.57%)
Materials and equipment were clean	97 (75.78%)	29 (22.66%)	2 (1.56%)	0	0
The temperature was comfortable	83 (64.84%)	39 (30.47%)	3 (2.34%)	1 (0.78%)	2 (1.56%)
The waiting time was acceptable	74 (59.2%)	44 (35.2%)	4 (3.2%)	2 (1.6%)	1 (0.8%)
The dental student was professional and showed his/her concern regarding my child’s treatment	101 (78.91%)	26 (20.31%)	1 (0.78%)	0	0
My questions had been answered	100 (79.37%)	26 (20.63%)	0	0	0
I am overall satisfied with the visit	96 (75.00%)	31 (24.21%)	0	1 (0.78%)	0

The results also showed that 62% of participants were seen on time or earlier, while the rest waited between 15 minutes and one hour. Waiting time was found to be statistically significantly associated with overall parental satisfaction (p = 0.002), as shown in Table 4.

**Table 4:** Associations between participant characteristics and overall parental satisfaction with pediatric dental visits (n = 128) *Percentages in the “Frequency” column represent the distribution of participants in each category out of the total sample (n = 128). **Percentages in the “Overall satisfaction” column represent the proportion of participants within each subgroup who responded “Agree” or “Strongly Agree” to the item: “I am overall satisfied with the visit.” Satisfaction was dichotomized as “Satisfied” (Agree/Strongly Agree) vs. “Not Satisfied” (Neutral/Disagree/Strongly Disagree). ***p-values were calculated using Fisher’s exact test to assess associations between each variable and overall satisfaction

Factors	Frequency (% of total, n = 128)*	Overall satisfaction (% within category)**	p-value***
Student's level			
D3	54 (42.19%)	100%	0.344
D4	74 (57.81%)	98.64%	
Parents			
Father	48 (37.50%)	100%	0.433
Mother	80 (62.50%)	98.75%	
Parent's age			
18-25	3 (2.34%)	100%	0.208
26-34	34 (26.56%)	100%	
35-44	74 (57.81%)	100%	
45-54	16 (12.50%)	93.75%	
55-66	1 (0.78%)	100%	
Educational level			
Uneducated	3 (2.34%)	100%	0.051
Primary school	6 (4.69%)	100%	
Intermediate school	9 (7.03%)	100%	
High school	43 (33.59%)	97.76%	
Bachelor's degree	61 (47.66%)	100%	
Higher education	6 (4.69%)	100%	
Reason for visit			
Full dental check-up	21 (16.41%)	100%	0.042
Tooth decay	46 (35.94%)	100%	
Swelling	1 (0.78%)	100%	
Pain	5 (3.91%)	100%	
Dental trauma	1 (0.78%)	100%	
Teeth alignment concerns	3 (2.34%)	33.33%	
Planned treatment	51 (39.84%)	100%	
Waiting time			
Seen on time or earlier	79 (61.72%)	100%	0.002
15 min	23 (17.97%)	100%	
15-30 min	10 (7.81%)	100%	
30-60 min	10 (7.81%)	90.00%	
>60 min	2 (1.56%)	100%	
Cannot remember	4 (3.13%)	100%	

In addition to high satisfaction scores across individual service aspects, significant associations were observed between overall parental satisfaction and two key variables: reason for visit (p = 0.042) and waiting time (p = 0.002). These relationships are summarized in Table 4. 

## Discussion

Patients' experiences and satisfaction are intricate measures of healthcare quality and processes [13]. The satisfaction level is implicit in compliance, continuity of care, and reported outcomes [5]. In the context of patients’ satisfaction with the performed dental care, the pediatric dentistry undergraduate curriculum at the COD, KSAU-HS is designed to provide high-quality dental education and high-standard clinical practice. The college emphasizes continuing education and professional development programs to achieve excellence in education, research, patient care, and assessment. The present study assessed pediatric dental patients’ parental satisfaction with the dental care provided by undergraduate dental students at the COD, KSAU-HS in Riyadh, Saudi Arabia. A self-developed questionnaire was structured to evaluate parental satisfaction in undergraduate dental clinics, measuring satisfaction across appointment scheduling, facilities, and treatment quality.

The number of participants in this study exceeded the estimated minimum sample size, and the response rate was relatively high, aligning with rates reported in comparable surveys [3,11,14-17]. The findings are broadly consistent with prior research examining parental satisfaction with pediatric dental care. In this study, satisfaction was analyzed in relation to demographic, social, and care-related factors.

While numerous studies have explored parental satisfaction in pediatric dental settings, the discussion here emphasizes three key studies conducted in similar academic teaching environments, where care was provided by undergraduate dental students. These were intentionally selected to ensure contextual alignment with the present study’s institutional setting and training structure. Our results closely align with those of Tashkandi et al., Balhaddad et al., and Ahmedani et al. [11,14-15], all of whom reported high levels of parental satisfaction, alongside variations in dissatisfaction rates across service domains.

Prior literature presents mixed findings regarding the association between demographic variables and satisfaction levels [3,11,14-17]. Some studies identified significant associations between demographic factors and satisfaction [11,17], while others did not [14,15]. For example, Balhaddad et al. reported significant associations with education level and age, attributing this to higher expectations among more educated parents [11]. In contrast, Tashkandi et al. found no significant associations, which may have been influenced by a limited age range within the study sample [14]. In our study, demographic and social factors were not significantly associated with overall parental satisfaction.

Factors related to appointment booking, treatment delivery, and clinical facilities are critical in evaluating pediatric dental care quality [5,12]. This study revealed high satisfaction levels in these areas. Parents expressed strong satisfaction with appointment feasibility (92.9%), accessibility of the college (96%), and acceptable waiting times (94%). These findings align with those of Ahmedani et al., who reported a 76.78% satisfaction rate for ease of appointment booking [15]. Conversely, Tashkandi et al. and Balhaddad et al. noted lower satisfaction levels regarding appointment scheduling and waiting time [11,14]. In our study, waiting time was a key factor influencing satisfaction. Table 3 shows a statistically significant association between satisfaction levels and waiting time (p = 0.002). Inglehart et al. similarly found that long wait times negatively impacted satisfaction and reduced the likelihood of patients returning or referring others [18]. In our sample, the only participant who reported overall dissatisfaction also expressed reluctance to recommend the service.

A statistically significant association was observed between the reason for visit and overall parental satisfaction (p = 0.042), with most visit categories, such as full dental check-ups, caries management, and treatment continuation, showing near-universal satisfaction rates. However, lower satisfaction was noted in a small subset of caregivers whose children presented for teeth alignment concerns, with only 33.3% reporting satisfaction. While this group comprised only three participants, the result may reflect heightened expectations related to aesthetic outcomes or the limitations of immediate treatment options for such concerns within an undergraduate teaching environment. Despite the statistical significance, the small sample size prevents definitive conclusions and suggests a need for further research into how treatment complexity and visit type influence satisfaction in pediatric dental care.

Further analysis revealed that many parents selected the COD undergraduate clinics due to perceived treatment quality. Satisfaction rates were high regarding treatment time (91.34%) and treatment outcomes (96%). Professionalism and communication skills of the students also received excellent ratings, with 99.22% of parents satisfied, and 100% agreeing that their questions were adequately answered. These findings are consistent with previous literature [11,14-15].

The quality of clinical facilities also contributed to parental satisfaction. Parents reported high satisfaction with the cleanliness of the waiting room (96%), toilet adequacy (90.55%), equipment cleanliness (98.44%), and temperature comfort (95.31%). Balhaddad et al. similarly reported that 87.4% of respondents found waiting rooms clean and 83.1% found equipment clean [11]. Ahmedani et al. reported 81.65% satisfaction with cleanliness and 63.75% with comfort [15]. In contrast, Tashkandi et al. found dissatisfaction related to parking and clinic temperature [14]. In our study, overall satisfaction with COD clinic services exceeded 99%, and 95% of parents indicated they would recommend the clinic to others.

The findings of this study contribute to the growing literature on parental satisfaction with pediatric dental care and support the need to improve patient-centered approaches in undergraduate dental training. Alqahtani and Alawaji reported that adherence to pediatric appointments at COD was the highest among all dental specialties (80.58%) [19]. These findings highlight the success of pediatric dental services at COD in delivering accessible, high-quality care that meets parental expectations.

This study offers several notable strengths. It addresses a relevant and underexplored area: parental satisfaction with pediatric dental care delivered by undergraduate students in a teaching hospital setting. The study used a previously validated, structured 30-item questionnaire adapted for local use, and content validity was re-established through expert review. Data collection was conducted through face-to-face administration by trained bilingual interns, enhancing response accuracy and minimizing missing data. The use of Fisher’s exact test ensured appropriate statistical analysis for small sample groups. Ethical approval was obtained, and participant confidentiality was strictly maintained.

Despite its contributions, this study has several limitations. The use of convenience sampling and the restriction to a single institutional setting may limit the generalizability of the findings to broader populations. Additionally, the nonrandomized sampling approach introduces the potential for sampling bias, reducing the representativeness of the results.

Although the overall sample size met the calculated requirement, the small size of specific subgroups, such as those presenting for trauma or orthodontic concerns, limited the statistical power of subgroup comparisons. The use of a five-point Likert scale, while practical, may not fully capture the complexity and nuance of caregiver perceptions.

Due to the study’s exploratory nature and sample size constraints, multivariate analysis to control for potential confounding variables was not conducted. Moreover, the study did not account for unmeasured factors such as prior dental experiences, caregiver expectations, or communication challenges, which may have influenced satisfaction responses.

While the questionnaire was adapted from a previously validated instrument, internal consistency metrics (e.g., Cronbach’s alpha) were not calculated in this study. However, content validity was reassessed by experts, and a pilot test was conducted to ensure clarity and usability.

To improve the external validity and analytical depth of future research, we recommend conducting multicenter studies across different regions in Saudi Arabia, employing probability-based sampling and multivariable analytical models with larger, more diverse samples.

## Conclusions

In conclusion, parental satisfaction is a critical component of evaluating the quality of pediatric dental care, particularly in academic clinical settings where undergraduate students provide treatment. This study assessed caregivers’ satisfaction across various dimensions, including appointment accessibility, communication, facility environment, and treatment outcomes. It also explored how satisfaction levels were influenced by contextual factors such as waiting time and reason for the visit.

The findings indicate that most caregivers reported high satisfaction with the care their children received from undergraduate dental students at this institution. These results are consistent with existing literature and underscore the importance of integrating patient-centered feedback into dental education and service delivery.

However, due to the cross-sectional design, convenience sampling, and single-institution setting, the generalizability of the results is limited. Additionally, the absence of multivariate adjustment for potential confounding variables warrants cautious interpretation. Future research should adopt broader, multicenter approaches and include more robust analytical models to further explore the determinants of parental satisfaction in pediatric dentistry. Continual assessment of caregiver satisfaction remains essential for guiding improvements in clinical training and ensuring the delivery of high-quality, patient-centered care in academic dental settings.
